# Inclusion-Body Myositis Associated with Alzheimer's Disease

**DOI:** 10.1155/2013/536231

**Published:** 2013-03-27

**Authors:** Danijela Levacic, Leema Reddy Peddareddygari, David Nochlin, Leroy R. Sharer, Raji P. Grewal

**Affiliations:** ^1^New Jersey Neuroscience Institute at JFK Medical Center, 65 James Street, Edison, NJ, USA; ^2^The Neurogenetics Foundation, Cranbury, NJ, USA; ^3^Division of Neuropathology, Department of Pathology and Laboratory Medicine, New Jersey Medical School, 185 South Orange Avenue, Newark, NJ, USA; ^4^Neuroscience Institute, Saint Francis Medical Center, 601 Hamilton Avenue, Trenton, NJ, USA

## Abstract

Sporadic inclusion-body myositis (s-IBM) is a myopathy that is characterized by progressive weakness and muscle pathology demonstrating inflammation and rimmed vacuoles. In addition, similar to the pathology observed in the brains of patients with Alzheimer's disease, the deposition of beta-amyloid and phosphorylated tau proteins in muscle fibers has been reported. These shared pathologic features have prompted hypotheses suggesting a shared etiology of these two conditions. We report a case of a 73-year-old woman initially diagnosed with s-IBM who later developed Alzheimer's disease.

## 1. Introduction 

Sporadic inclusion-body myositis (s-IBM) is a muscle disease which presents with slowly progressive proximal and distal weakness. Clinically, it is characterized by early onset weakness and atrophy of the quadriceps, finger flexors and foot dorsiflexors [1]. It is the most common myopathy in individuals over age 50 years [2]. The diagnosis is confirmed by characteristic findings on muscle biopsy demonstrating endomysial inflammation, eosinophilic cytoplasmic inclusions, and rimmed vacuoles. The cause for s-IBM is not known; however a number of pathophysiologic mechanisms have been proposed. These include overexpression of amyloid beta precursor protein (APP), deposition of *β*-amyloid, C- and N-terminal APP fragments, hyperphosphorylation of tau proteins, abnormal accumulation of components related to lipid metabolism, oxidative stress, abnormal signal transduction, transcription and RNA accumulation, and lymphocytic inflammation [3–7]. Some of these pathophysiological mechanisms and pathologic abnormalities are similar to those observed in the brains of patients with Alzheimer's disease [3, 7]. This observation has led to theories of a shared pathophysiology between these two disparate disorders [7]. There is a precedent for brain and muscle involvement in one disorder: inclusion-body myopathy, Paget's disease of the bone, and frontotemporal dementia (IBMPFD) [8]. In this autosomal dominant disease, inclusion-body myopathy is associated with frontotemporal dementia and is caused by mutations in the *valosin containing protein* gene. This condition confirms that mutation of a shared protein can result in disease affecting both brain and muscle tissues. 

In spite of the high prevalence of Alzheimer's disease, there are few reports of patients with coexisting s-IBM [2]. We report a patient who initially presented with symptoms and signs consistent with s-IBM and subsequently developed Alzheimer's disease.

## 2. Case Presentation

 A 73-year-old woman presented to Neuromuscular Clinic at the Neuroscience Institute/JFK Hospital, Edison, New Jersey, USA, with complaints of weakness. She had difficulty climbing up and down stairs, arising from a deep-seated chair and using her arms above her head. These symptoms had slowly progressed over 5 years prior to presentation. There were no complaints of sensory loss, difficulty speaking, chewing, swallowing, diplopia, or cognitive complaints.

 Her neurological examination showed a normal mental status and cranial nerve examination. Her deep tendon reflexes were symmetrical: +2/4 in upper extremities, +1/4 at the patellae, and unobtainable at the ankles. Her plantar reflexes were flexor. Muscle strength testing showed diffuse weakness (MRC grade 4/5) testing the following: neck flexion, wrist flexion and extension, biceps, triceps, supraspinatus, and internal and external rotation. The most profound upper extremity weakness was testing the finger flexors. She could not make a fist. She had 4/5 weakness of hip flexion and 3/5 weakness of the tibialis anterior muscles bilaterally. Atrophy was noted of the forearms, quadriceps, and tibialis anterior muscles bilaterally. Sensory examination disclosed normal light touch, pin prick, vibration, and proprioception in her arms and legs. She had no tremor or ataxia. She was unable to tandem walk or walk on her heels or toes.

Routine serum chemistries were normal except for a CPK of 470 (normal < 165) and an aldolase of 12.4 (normal range 0–7). 

Electrophysiological testing showed normal nerve conduction parameters in all motor (ulnar, median, personal and tibial) and sensory (ulnar, median, radial, superficial, and sural) nerves tested. Needle electromyogram showed increased irritability but no abnormal spontaneous activity in any muscle sampled. Further analysis disclosed low amplitude motor units, early recruitment, and a full interference pattern with submaximal effort. Overall, the study was consistent with a myopathy with minimal inflammatory features.

**Figure 1:**
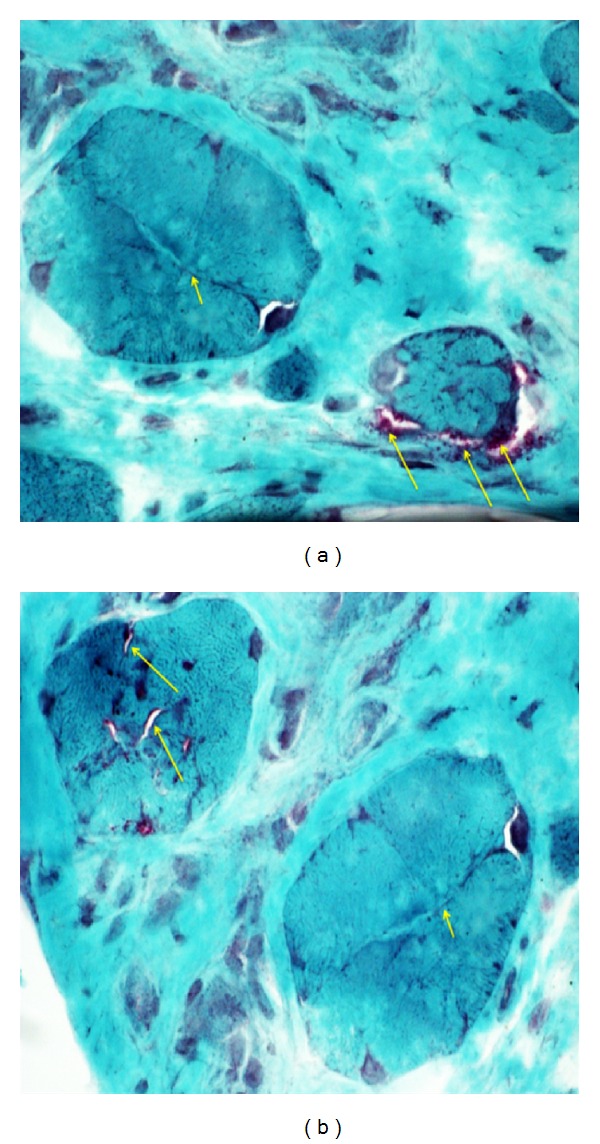
(a) Muscle fiber showing rimmed vacuoles (granular, red) (long arrows), and fiber splitting (short arrow). (b) Muscle fiber showing slit-like rimmed vacuoles (Long arrows), with fiber splitting (short arrow). Histochemistry, section of anterior tibialis muscle stained with Engel's modified Gomori trichrome stain. Isopentane cooled in liquid nitrogen frozen section. 25× original magnification.

A muscle biopsy of the right tibialis anterior muscle was performed and showed scattered rimmed vacuoles in the end-stage muscle, compatible with a diagnosis of inclusion-body myositis (Figure 1).

She was treated for 6 months with IVIG (2 grams/kilogram) by slow intravenous infusion on a monthly basis. Although she felt an increase in strength, there was no objective evidence of improvement and it was ultimately discontinued. 

Approximately three years after initial diagnosis, she started to develop symptoms of cognitive decline with difficulties in memory, speech, and attention. Reexamination five years after the initial evaluation showed she was disoriented to time, place, and person and could not follow a 2-step command. She was not able to calculate and had poor attention. Her tests of short- and long-term memory were impaired. Further cognitive testing revealed transcortical sensory aphasia, frontal lobe dysfunction with reduced fluency and perseveration. Investigations for dementia were performed and the following tests were normal or negative: thyroid function studies, rapid plasma reagin, and serum vitamin B12. A magnetic resonance imaging of the brain disclosed moderate atrophy with no evidence of multiple infarcts or hydrocephalus. 

A neuromuscular reexamination performed at this time showed increased weakness of the same muscle groups as documented in the prior examination.

## 3. Discussion

The prevalence of s-IBM has been estimated at 51 per million in people over age 50 years. In an observational study of 35 s-IBM patients the age adjusted prevalence rate for individuals over the age 45 years was 28.9 × 10^−6^. The mean age at onset was 70.0 years with only two patients who developed weakness before age 50 years [9]. The age adjusted prevalence of Alzheimer's disease is reported as 4.4% in people over the age of 65 years. This frequency increases with each decade with a nearly fifteen-fold increase in the prevalence in individuals between 60 and 85 years [10]. In spite of the high frequency of Alzheimer' disease, it is interesting to note that there is only one report of a patient with both of these disorders. This is a case of a 65-year-old woman who presented with the symptoms and signs consistent with s-IBM and the subsequent diagnosis of Alzheimer's disease [2]. 

There are several similarities between our patient and the subject of the previous report. Both are women in whom the initial presentation and diagnosis were those of s-IBM with the subsequent development of Alzheimer's disease. The presentation in these patients may provide a clue to the infrequency of the reported association of these two disorders. Although both disorders do show an increase in incidence with age, Alzheimer's disease is more frequent than s-IBM. This initial diagnosis of Alzheimer's disease could result in decreased chance of a subsequent diagnosis of s-IBM for several reasons. Firstly as cognitive decline develops, the patients themselves may be less likely to voice any complaints of muscle weakness. Secondly as the cognitive decline continues, the patients become less mobile and medical caregivers may ascribe the development of atrophy or weakness to deconditioning rather than coexisting muscle disease. 

A number of theories have been proposed indicating that Alzheimer's disease and s-IBM share a similar pathogenesis. Whether these two cases support these theories or represent a chance association will be confirmed by further studies. Such studies are important as they will not only provide clues to the development of both disorders but also may facilitate the development of shared therapeutic agents.
